# Intra-patient intra-tumoral immune heterogeneity is evident at progression on targeted therapy and immunotherapy for melanoma

**DOI:** 10.1186/2051-1426-2-S3-P200

**Published:** 2014-11-06

**Authors:** Alexandre Reuben, Zachary A Cooper, Rodabe N Amaria, Marie A Forget, Cara Haymaker, Chantale Bernatchez, Pei-ling Chen, Michael T Tetzlaff, Lynda Chin, Karen C Dwyer, Jennifer A Wargo

**Affiliations:** 1 Surgical Oncology, MD Anderson Cancer Center, Houston, TX, USA; 2 Genomic Medicine, MD Anderson Cancer Center, Houston, TX, USA; 3 Melanoma Medical Oncology, MD Anderson Cancer Center, Houston, TX, USA; 4 Pathology, MD Anderson Cancer Center, Houston, TX, USA; 5 Stem Cell Transplantation, MD Anderson Cancer Center, Houston, TX, USA

## 

There have been significant advances in the treatment of melanoma via the use of targeted therapy and immunotherapy, however a significant proportion of patients still progress on treatment. Intense research efforts are underway to better understand resistance, and multiple molecular resistance mechanisms to targeted therapy have been identified. There is a growing appreciation of intra-tumoral genetic heterogeneity as a contributor to resistance to therapy, however immune heterogeneity within multiple solid tumors within the same patient has been less well studied. The goal of the present study is to better understand the leukocyte infiltrate in multiple melanoma tumors within the same patient at the time of disease progression, with the potential to identify actionable strategies to overcome resistance to therapy. In these studies, we prospectively evaluated 8 tumors from 4 patients progressing on either targeted therapy or immunotherapy. Tissues were evaluated by polychromatic flow cytometry in combination with immunohistochemical analysis of tissue sections. Specifically, we utilized 5 panels of antibodies for flow cytometry to gain a deep analysis of the leukocyte composition with a focus of 1) T cells/Tregs, 2) NK/T/B cells, 3) tumor associated macrophages and fibroblasts, 4) dendritic cells, and 5) polymorphonuclear leukocytes. Results demonstrate significant immune heterogeneity between different melanoma tumors within the same patient in the majority of patients studied (3 of 4). Of note, there were significant differences in CD4+, CD8+, and regulatory T cells (p < 0.05) with similar activation markers but differences in T cell memory markers. There were no significant differences in tumor-associated macrophages or dendritic cells. Together, these data suggest that there may be significant immune heterogeneity between different tumors within a single patient with metastatic melanoma. This has important clinical implications, as a single tumor biopsy sample may not be representative of the immune profile of multiple tumors within the same individual. It is provocative to contemplate that this could account for variable responses to therapy, however this is a hypothesis that needs to be tested carefully in a much larger data set. Nonetheless, these findings have potential significant implications for the treatment of melanoma and other cancers.

